# One health approach to *Rickettsia* spp.: Brazilian indigenous individuals, their dogs and ticks, and healthcare professionals

**DOI:** 10.1016/j.onehlt.2025.101025

**Published:** 2025-04-03

**Authors:** Louise Bach Kmetiuk, Vamilton Alvares Santarém, Daniele Rodrigues, Suelen Teixeira de Faria Resende, Isabella Braghin Ferreira, Rogério Giuffrida, Bianca Bárbara Fonseca da Silva, Lucianne Cardoso Neves, Raphaela Bueno Mendes Bittencourt, Leandro Meneguelli Biondo, Fabiano Borges Figueiredo, Felipe da Silva Krawczak, Alexander Welker Biondo

**Affiliations:** a Municipal Secretary of Health, Curitiba, PR, Brazil; b Graduate College in Animal Sciences, University of Western São Paulo (UNOESTE), Presidente Prudente, SP, Brazil; c Special Indigenous Health District South Coast (DSEI), Brazilian Ministry of Health, Curitiba, PR, Brazil; d School of Veterinary Medicine and Animal Science, Federal University of Goiás, GO, Brazil; e National Institute of the Atlantic Forest (INMA), Brazilian Ministry of Science, Technology, and Innovation, Santa Teresa, ES, Brazil; f Carlos Chagas Institute, Oswaldo Cruz Foundation, Curitiba, PR, Brazil; g Department of Veterinary Medicine, School of Veterinary Medicine, Federal University of Paraná, Curitiba, PR, Brazil

**Keywords:** One health, Tick-borne rickettsiae, Human, Dogs

## Abstract

Although Indigenous populations have historically overlapped the occurrence of vector-borne pathogens, no One Health approach study has investigated *Rickettsia* spp. in indigenous communities worldwide. Accordingly, the aim of this study was to investigate anti-*Rickettsia* spp. antibodies in indigenous individuals, their dogs and healthcare professionals, and *Rickettsia* spp. infection in ticks from ten indigenous communities of southern and southeastern Brazil. In overall, 66/771 (8.6 %) indigenous individuals, 9/99 (9.1 %) healthcare professionals and 116/386 (30.1 %) dogs were seropositive for at least one out four *Rickettsia* species tested by immunofluorescence assay (IFA). Out of 603 ticks collected from dogs in indigenous communities, 9/190 (4.7 %) tested positive to fragment of *Rickettsia gltA* gene by real-time PCR. The homologous antigenic reactions in dogs were significantly more frequent for *R. bellii* when compared to *R. parkeri* and *R. amblyommatis* and may be associated with the high diversity of hard and soft ticks in Americas, and *R. bellii* capacity of inhibiting another pathogenic rickettsia. Tick bite history increased the seropositivity (odds ratio = 9.29; *p* = 0.019) in healthcare professionals. This difference may be consequence of higher capacity to recognize tick bites by healthcare professionals, which highlighted the necessity of health care education for indigenous individuals for prevention and early recognition of tick-borne diseases in indigenous communities. In addition, the One Health approach herein has provided a holistic understanding of *Rickettsia* spp. infection in such communities and correspondent healthcare personal.

## 1 Introduction

The *Rickettsia* genus has been an obligately intracellular gram-negative bacteria causing zoonosis worldwide, mainly transmitted by ticks [1]. Humans rickettsiosis cases in Brazil has been mostly caused by rickettsiae belonging to Spotted Fever Group (SFG) and showed different clinical features, including *Rickettsia rickettsii* with high case-fatality rate, *Rickettsia parkeri* strain Atlantic Rainforest and *Rickettsia parkeri* sensu stricto, associated with milder non-lethal febrile disease and inoculation eschar [2,3].Despite human beings have been considered less exposed to ticks (and therefore rickettsiae) than animals, specific human settings and activities such as hunting and living next natural areas may increase the exposure risk to tick-borne diseases [4].

Indigenous populations worldwide have been lived intrinsically interconnected with nature [5]. Although historically endangering human survival by predation and (direct and vector-borne) diseases, wildlife fauna has provided vital animal-based protein for human perseverance [6]. Such perception of human-animal health interconnectedness can be traced within the traditional culture and understanding of indigenous peoples in the Americas, long before European invasion. Despite wildlife domestication into livestock may apparently facilitate protein availability, not a single native animal species was domesticated by indigenous peoples in Brazil during their 20–30,000 years of endurance [7]. Interestingly, only six animal species were found domesticated in pre-Columbian America, including two large birds (turkey and Muscovy duck) in North in Central America, and four Andean species: two camelids (llama and alpaca) and two medium-sized rodents (guinea pig and chinchilla) [8]. In such a scenario, exotic companion (dogs and cats) and livestock (cattle, horses, swine, goats and sheep) animal species brought to America and currently living in Indigenous communities throughout Brazil may have impacted the long-lasting One Health balance, providing favorable conditions for emergent, reemergent and novel disease cycles.

In Brazil, indigenous populations were estimated in 1693,535 individuals in 2022, which represented approximately 0.83 % of the general population, according to national census at the time [9]. As suggested by a previous study, indigenous territories and populations have been historically overlapped with vector-borne diseases occurrence worldwide [10], associated with lowest government investment in health per country and low income in these tropical and subtropical areas [10]. In addition, as mentioned, indigenous communities have modified their environment after European invasion with hunting dogs, livestock and agriculture practices, bringing together wildlife, domestic animals and community, increasing blood-supply to ticks and exposure to tick-borne rickettsiae diseases [11].

Although few studies have assessed indigenous populations worldwide, the risk of exposure to *Rickettsia* spp. belonging to the spotted fever group with fatal cases in indigenous populations has only been reported in Central America [12]. In Colombia, 141/539 (26.2 %) individuals from indigenous communities were seropositive to *Rickettsia* spp. of Spotted Fever Group (SFG), with higher seropositivity associated to close livestock contact [13]. In Panama, *Rickettsia rickettsii* was isolated from an outbreak in a remote indigenous community, without recent tick bite history [14]. In the Peninsular Malaysia, indigenous individuals and animal farm workers presented similar and higher seropositivity to *Rickettsia* spp. of SFG than blood donors of urban areas, along with positive cattle ticks [15]. In the central-western region of Brazilian Amazon, 16/36 (44.4 %) tick and no/121 louse DNA pools collected from asymptomatic indigenous individuals were infected by *Rickettsia* spp., warning for the importance of febrile outbreaks in such areas [16].

Despite human and dog populations living in indigenous communities may be exposed to tick-borne rickettsiae, no study to date has concurrently assessed *Rickettsia* spp. in indigenous people, their dogs, and healthcare professionals working in indigenous communities. Moreover, rickettsiosis outbreaks (Rocky Mountain spotted fever) leading to fatal cases in indigenous communities have been reported in Central America [12], highlighting the importance of *Rickettsia* spp. surveys in these communities. Accordingly, the present study aimed to assess the exposure of indigenous individuals, their dogs and health professionals to ticks and rickettsial agents, along with associated risk factors, in ten indigenous communities located in the southern and southeastern regions of Brazil.

## 2 Material and methods

### 2.1 Ethics

The present study has been approved by the National Human Ethics Research Committee (protocol 52039021.9.0000.0102) and by the Ethics Committee of Animal Use (protocol number 033/2021) at the Federal University of Paraná.

### 2.2 Study design and area

This is a cross-sectional seroepidemiological study on *Rickettsia* spp. exposure in ten indigenous communities of Paraná and São Paulo States, located in southern and southeastern Brazil, respectively. Indigenous individuals, their dogs and healthcare professionals were assessed for *Rickettsia* spp. seroprevalence and associated risk factors. In addition, ticks infesting dogs were collected, identified, and molecularly analyzed for *Rickettsia* spp. infection.

### 2.3 Sample collection

Indigenous participants from Guarani, Terena, and Kaingang ethnicities were sampled in ten indigenous communities of Paraná and São Paulo States from December 2020 through July 2022, which included summer, autumn and winter. The study was conducted in both preserved and degraded areas in the Atlantic Forest biome of southern Brazil, including seashore and countryside municipalities; and in degraded areas of Cerrado biome in southeastern Brazil, including countryside municipalities.

### 2.4 Serum samples

Human samples were collected by certified nurses via cephalic venipuncture, after completing the consent form and an epidemiological questionnaire. Dog samples were collected by certified veterinarians via jugular venipuncture after the consent form and an epidemiological questionnaire completed by owner. All blood samples were placed in tubes without anticoagulant, kept at room temperature until visible clots formed, centrifuged at 1,500 RPM for five minutes, serum separated and kept at −20 °C until processing.

### 2.5 Tick collection

Dogs were carefully examined for tick presence after blood collection. All ticks obtained were preserved in isopropyl alcohol and taken to the laboratory for taxonomic identification, which was performed following standard morphological keys [17].

#### 2.5.1 Detection of anti-Rickettsia spp. antibodies

Sera from dogs and humans were tested using an immunofluorescence assay (IFA) targeting four *Rickettsia* antigens isolated from Brazil: *R. rickettsia* strain Pampulha, *Rickettsia parkeri* strain Atlantic rainforest, *Rickettsia amblyommatis* strain Ac37, and *Rickettsia bellii* strain Mogi, as previously described [18]. Briefly, sera were diluted in two-fold increments with phosphate-buffered saline (PBS) from an initial dilution of 1:64. The slides were incubated with fluorescein isothiocyanate-labelled rabbit anti-dog IgG (Sigma, St Louis, MO, USA) and rabbit anti-human IgG (IgG, Sigma, St. Louis, MO, USA) for canine and human sera, respectively. For each sample, the endpoint IgG titer reacting with each of the four *Rickettsia* antigens was determined. An endpoint titer at least four-fold higher for a *Rickettsia* species than those observed for the other *Rickettsia* species was considered probably homologous to the first *Rickettsia* species or a very closely related species [19]. On each slide, a non-reactive serum (negative control) and reactive serum (positive control) from dogs or humans from the other studies were tested at a 1:64 dilution [18,20].

#### 2.5.2 Molecular detection of Rickettsia spp. in ticks

Ticks collected from dogs were randomly selected and individually processed for DNA extraction, using the guanidine isothiocyanate and phenol/chloroform technique [21]. DNA from ticks were tested with a TaqMan real-time qPCR assay targeting a 147 bp fragment of the rickettsial citrate synthase (*gltA*) gene [22]. The qPCR positive samples were additionally tested using a conventional PCR assay targeting a 532 bp fragment of the 190-kDa outer membrane protein gene (*ompA*) of the spotted fever group (SFG) *Rickettsia* spp. [23]. PCR-negative samples were further tested using PCR protocols targeting the *16S rDNA* gene of ticks [24] to validate the tick-DNA extraction protocol. If a sample did not produce any product in these PCR assay, the sample was discarded.

PCR products for the *ompA* and *16S rDNA* genes were stained with SYBR Safe (Invitrogen, Carlsbad, CA, EUA), according to the manufacturer's recommendations, and visualized by electrophoresis in a 1.5 % agarose gel using an ultraviolet transilluminator.

### 2.6 Epidemiological data collection

Epidemiological analysis of human associated risk factors for ticks and *Rickettsia* spp. exposure were based on epidemiological questionnaire, and included indigenous community location, age, gender, education level, occupation, family members at the household, time living in the area, animal ownership, frequency of contact with forest areas, previous tick bite, and location of tick contact (household, after contact with forest area, other). Epidemiological analysis of dog characteristics was based on a questionnaire that assessed potential associated risk factors to ticks and *Rickettsia* spp. and included the sex, breed, animal origin, access to natural areas, hunting habit, and tick sampling. The data were organized in spreadsheets, and the analytical process started with a descriptive exploration of the databases.

### 2.7 Statistical analysis

The Pearson's Chi-square or Fisher's exact test was used to evaluate the association between the seropositivity for *Rickettsia* spp. in indigenous (*n* = 666), healthcare professionals (*n* = 99) and dogs (*n* = 376), considering the epidemiological gathered information. Odds ratio and 95 % confidence interval (95 % CI) were calculated for the associations. Chi-square was performed to evaluate the difference between the seropositivity proportions in indigenous and healthcare professionals. Cochran's Q Test was applied to compare dog and human seropositivity (≥ 1:64) of four *Rickettsia* species tested, regardless location and possible antigen responsible for the infection. Alternatively, non-homologous seropositivity between *Rickettsia* species was also compared using Cochran's Q Test. Paired comparison was evaluated by McNemar's Chi-Square Test with significant level corrected by Bonferroni Method. The Chi-Square Test was used to compare proportions of seropositivity between Paraná and São Paulo State for each *Rickettsia* spp. tested. Homologous reactions of *R. parkeri*, *R. amblyommatis* and *R. belli* were tested by Chi-Square Test regardless location. All statistical analyses were conducted in R software v. 4.2.3 (R core team), with a *P*-value of <0.05 considered significant.

## 3 Results

**Fig. 1:**
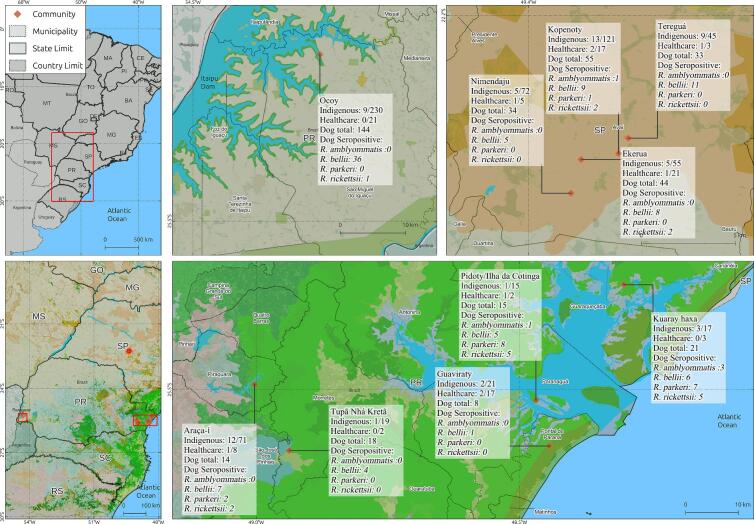
Sampling location and distribution of *Rickettsia* spp. seropositivity in indigenous individuals of Brazil.

In overall, seropositivity for at least one rickettsial antigens was observed in 66/771 (8.6 %; 95 % CI: 6.8–10.8 %) indigenous, 116/386 (30.1 %; 95 % CI: 25.7–34.8 %) dogs and 9/99 (9.1 %; 95 % CI: 4.9–16.9 %) healthcare professionals. Comparison of proportions for seropositivity in indigenous and healthcare professionals was not statistically significant (Chi-square = 0.031; *p*-value = 0.859). In general, human samples from indigenous and healthcare professionals have shown a homologous seropositive reaction of 6.1 % (4/66), 6.1 % (4/66) and 4.5 % (3/66) in the IFA as probable homologous antigen to *R. parkeri*, *R. amblyommatis* and *R. bellii*, respectively. No significant differences (χ^2^ = 0.80, df = 2, *p* = 0.669) were found for human samples among the proportions of homologous antigenic reactions of *R. bellii* (0.26 %), *R. parkeri* (0.52 %), and *R. amblyommatis* (0.52 %) (Fig. 1; Table 1).

**Table 1:** Seroreactivity to four *Rickettsia* species of human and dog samples from ten indigenous communities of southern and southeastern, Brazil.

Study area	Samples (n seropositive/n tested)	Number of seroreactive Human or Dog to each of the *Ricketttsia* species/ % seroreactivity for Human or Dog (range of endpoint titer)				No. samples with determined homologous reaction (PAIHR)⁎
		*R rickettsii*	*R. parkeri*	*R. amblyommatis*	*R. bellii*	
Araça-í	Human (15/82)	7/8.5 (64–128)	8/9.7 (64–256)	7/8.5 (64–128)	7/8.5 (64–128)	2 (*R. parkeri*) 1 (*R. bellii*)
	Dog (8/14)	2/14.3(256)	2/14.3 (512–2048)	1/7.1 (64)	7/50 (128–512)	1 (*R. parkeri*) 6 (*R. bellii*)
Deuses da Montanha	Human (1/21)	1/4.8 (64)	0/0	1/4.8 (128)	0/0	–
	Dog (5/18)	0/0	0/0	0/0	5/27.7 (64–152)	4 (*R. bellii*)
Ekerua	Human (6/79)	1/1.3 (64)	0/0	4/5.1 (64–128)	2/2.5 (64)	2 (*R. amblyommatis*)
	Dog (15/44)	2/4.5 (64–128)	4/9.1 (64)	0/0	13/29.5 (64–512)	8 (*R. bellii*)
Guaviraty	Human (5/39)	1/2.6 (128)	5/12.8 (64–256)	3/7.7 (64)	0/0	2 (*R. parkeri*)
	Dog (1/8)	0/0	0/0	0/0	1/12.5 (256)	1 (*R. bellii*)
Kopenoty	Human (15/141)	6/4.2 (64–128)	12/8.5 (64–128)	10/7.1 (64–256)	2/1.4 (64–256)	–
	Dog (11/55)	2/3.6 (64)	1/1.8 (128)	1/1.8 (256)	10/18.2 (64–1024)	8 (*R. bellii*)
Kuaray Haxa	Human (3/21)	0/0	2/9.5 (64)	1/4.8 (64)	1/4.8 (64)	–
	Dog (10/21)	5/23.8 (64–512)	8/38.0 (64–1024)	4/19.0 (64–512)	7/33.3 (64–512)	1 (*R. parkeri*) 2 (*R. bellii*)
Nimendaju	Human (6/81)	2/2.5 (64)	0/0	3/3.7 (64–128)	1/1.2 (64)	1 (*R. amblyommatis*)
	Dog (6/34)	0/0	0/0	0/0	6/17.6 (64–512)	5 (*R. bellii*)
Ocoy	Human (12/341)	3/0.9 (64)	3/0.9 (64)	7/2.0 (64)	2/0.6 (64–128)	1 (*R. bellii*)
	Dog (38/144)	1/0.7 (128)	1/0.7 (64)	0/0	38/26.4 (64–1024)	36 (*R. bellii*)
Pidoty	Human (2/17)	0/0	0/0	0/0	2/11.8 (64–128)	1 (*R. bellii*)
	Dog (10/15)	5/33.3 (64–1024)	8/53.3 (256–2048)	3/20.0 (64–256)	5/33.3 (256–512)	4 (*R. parkeri*) 2 (*R. bellii*)
Teregua	Human (10/48)	2/4.2 (64)	6/12.5 (64–128)	7/14.6 (64–128)	2/4.2 (64–128)	1 (*R. amblyommatis*)
	Dog (12/33)	0/0	0/0	0/0	12/36.4 (64–512)	11 (*R. bellii*)
Total	Human (75/870)	23/2.6 (64–128)	36/4.1 (64–256)	43/4.9 (64–256)	19/2.2 (64–256)	4 (*R. parkeri*) 4 (*R. amblyommatis*) 3 (*R. bellii*)
	Dog (116/386)	17/4.4 (64–1024)	24/6.2 (64–2048)	9/2.3 (64–512)	104/26.9 (64–1024)	6 (*R. parkeri*) 83 (*R. bellii*)

⁎ A homologous reaction was determined when an endpoint titer to *Rickettsia* species was at least fourfold higher than those observed for the other *Rickettsia* species. In this case, the *Rickettsia* species (or a very closely related species) involved in the highest endpoint titer was considered the possible antigen involved in a homologous reaction (PAIHR).

No associated risk factor for human exposure to rickettsial agents (including living area, age, gender, hunting, tick bite history, and having at least a dog) was associated to indigenous individuals (Table 2). Tick bite history increased the seropositivity (odds ratio = 9.29; *p* = 0.019) in healthcare professionals, while other variables (gender, age, and working regimen in communities) were not associated with *Rickettsia* spp. seropositivity (Table 3).

**Table 2:** Associated risk factors for *Rickettsia* spp. seropositivity in indigenous from ten communities of southern and southeastern, Brazil.

	Seropositive (%)	Seronegative (%)	Univariate Analysis	
Characteristics	n = 60	*n* = 606	OR (95 % CI)	*p*
Location (State)				0.164
Paraná	28 (46.7)	345 (56.9)	1.0 [Reference]	
São Paulo	32 (53.3)	261 (43.1)	1.51 (0.88–2.59)	
Gender				0.745
Female	32 (53.3)	342 (56.4)	1.0 [Reference]	
Male	28 (46.7)	264 (43.6)	1.13 (0.66–1.93)	
Age (years)				0.219
up to 15	14 (23.3)	176 (29.4)	1.0 [Reference]	
16 to 23	16 (26.7)	131 (21.9)	1.53 (0.72–3.31)	
24 to 37	10 (16.7)	146 (24.4)	0.87 (0.36–2.01)	
> 37	20 (33.3)	145 (24.2)	1.73 (0.84–3.62)	
Hunting				0.786
No	48 (80.0)	470 (77.6)	1.0 [Reference]	
Yes	12 (20.0)	136 (22.4)	0.87 (0.43–1.64)	
Tick bite history				0.085
No	38 (77.6)	271 (64.1)	1.0 [Reference]	
Yes	11 (22.4)	152 (35.9)	0.52 (0.25–1.02)	
Having dog(s)				0.839
No	10 (16.7)	113 (18.6)	1.0 [Reference]	
Yes	50 (83.3)	493 (81.4)	1.13 (0.58–2.44)

**Table 3:** Associated risk factors for *Rickettsia* spp. seropositivity in healthcare professionals from ten indigenous communities of southern and southeastern, Brazil.

	Seropositive (%)	Seronegative (%)	Univariate Analysis	
Characteristics	n = 9	*n* = 90	OR (95 % CI)	*p*
Gender				0.736
Female	5 (55.6)	55 (61.1)	1.0 [Reference]	
Male	4 (44.4)	35 (38.9)	1.26 (0.28–5.28)	
Age (years)				0.492
21 to 36	6 (66.7)	46 (51.1)	1.0 [Reference]	
37 to 65	3 (33.3)	44 (48.9)	0.54 (0.10–2.25)	
Working regimen in communities				0.907
Up to 20 h/week	5 (55.6)	41 (45.6)	1.0 [Reference]	
20 to 40 h/week	2 (22.2)	23 (25.6)	0.74 (0.09–3.94)	
Sporadically (once a semester)	2 (22.2)	26 (28.9)	0.66 (0.08–3.47)	
Tick bite history				0.012
No	1 (11.1)	49 (57.0)	1.0 [Reference]	
Yes	8 (88.9)	37 (43.0)	9.29 (1.56–241)	

Likewise, no associated risk factor for dog exposure to rickettsial agents (including age, sex, hunting under supervision, and living area) was associated to a seropositive test (Table 4). Dog samples showed seropositive homologous reaction of 5.2 % (6/116) and 71.5 % (83/116) in the IFA as probable homologous antigen to *R. parkeri* and *R. bellii*, respectively (Table 1). Homologous antigenic reactions in dogs were significantly more frequent to *R. bellii* (79.8 %) when compared to *R. parkeri* (25 %) and *R. amblyommatis* (0 %) (χ^2^ = 25.12, df = 1, *p* < 0.001).

**Table 4:** Associated risk factors for *Rickettsia* spp. seropositivity in dogs from ten indigenous communities of Brazil.

	Positive (%)	Negative (%)	Univariate Analysis	
Characteristics	*n* = 112	*n* = 264	OR (95 % CI)	*p*
Age (years)				0.795
Up to 1	22 (25.0)	48 (23.3)	1.0 [Reference]	
2 to 7	65 (73.9)	152 (73.8)	0.93 (0.52–1.69)	
8 to 12	1 (1.14)	6 (2.91)	0.41 (0.02–2.69)	
Gender				0.143
Female	53 (52.0)	101 (42.6)	1.0 [Reference	
Male	49 (48.0)	136 (57.4)	0.69 (0.43–1.10)	
Hunting under supervision				0.119
No	77 (75.5)	157 (66.2)	1.0 [Reference]	
Yes	25 (24.5)	80 (33.8)	0.64 (0.37–1.07)	
Living area				0.261
Southern	68 (60.7)	142 (53.8)	1.0 [Reference]	
Southeastern	44 (39.3)	122 (46.2)	0.75 (0.48–1.18)	

A total of 603 ticks were collected from dogs of indigenous communities, including 74.3 % (448/603) adults of *Rhipicephalus sanguineus* s. l., 14.9 % (90/603) adults of *Amblyomma aureolatum*, 7.4 % (45/603) nymph of *Rhipicephalus* spp., 0.2 % (1/603) nymph of *Amblyomma* spp. and 3.1 % (19/603) adults of *Amblyomma ovale* (Table 5). Ticks [*n* = 190; 31.5 % (190/603)] were randomly selected for qPCR testing (Table 5), with 4.7 % (9/190) testing positive for the gltA gene amplification and no positive result for the ompA gene amplification. Positive ticks included 3.1 % (4/127) of *Rhipicephalus sanguineus* s.l., 7.7 % (1/13) of *Amblyomma ovale*, and 8 % (4/50) of *Amblyomma aureolatum*. All positive *A. aureolatum* and *A. ovale* to rickettsial DNA were from the Araça-í indigenous community, while 3.3 % (2/61) positive *Rhipicephalus sanguineus* s.l were from the Kopenoty and 5.9 % (2/34) from the Nimendaju communities. Although the cPCR for the *gltA* gene was performed to identify which rickettsial species may have infected the sampled ticks [25], sequencing was not performed due to low amplicon quality. A total of 2/9 (22.2 %) positive ticks were collected from seropositive dogs, and 4 out 5 remaining positive ticks were collected from not tested dogs. All negative results by qPCR for *Rickettsia* spp. were ensured by positive amplification of the *16S rDNA* constitutive (housekeeping) gene. (See Table 6.)

**Table 5:** Number and species of ticks collected from dogs and qPCR-positive ticks of ten indigenous communities of southern and southeastern, Brazil, from December 2020 to February 2022.

Study village	No. of dogs with ticks/No. of sampled dogs (% infested animals)	No. of ticks by species					
		*A. aureolatum*	*A. ovale*	*Amblyomma* spp.	*Rhipicephalus* spp.	*R. sanguineus* s.l.	No. of *gltA* qPCR-positive ticks/No. of samples (% of positives)
Araça-í	6/14 (42,8 %)	28 M, 31 F	10 M, 9 F	1 N	–	–	4/29 (13.8 %) *A. aureolatum;*1/13 (7.7 %) *A. ovale*
Deuses da Montanha	5/18 (27,8 %)	17 M, 14 F	–	–	–	–	0/21 (0) *A. aureolatum*
Ekerua	29/44 (65,9 %)	–	–	–	20 N	42 M, 46 F	0/26 (0) *R. sanguineus* s.l.
Guaviraty	n.a.	–	–	–	–	–	n.a.
Kopenoty	37/55 (67,3 %)	–	–	–	6 N	92 M, 115 F	2/61 (3.3 %) *R. sanguineus* s.l.
Kuaray Haxa	n.a.	–	–	–	–	–	n.a.
Nimendaju	24/34 (70,5 %)	–	–	–	12 N	68 M, 61 F	2/34 (5.9 %) *R. sanguineus* s.l.
Ocoy	n.a.	–	–	–	–	–	n.a.
Pidoty	n.a.	–	–	–	–	–	n.a.
Teregua	14/33 (42,4 %)	–	–	–	7 N	10 M, 14 F	0/6 (0) *R. sanguineus* s.l.
Total	115/198 (58,1 %)	45 M, 45 F	10 M, 9 F	1 N	45 N	212 M, 236 F	9/190 (4.7 %)

M: males; F: females; N: nymphs; (−): absence of ixodid; n.a.: not applicable.

**Table 6:** Frequency of tick species collected from dogs and prevalence of positive results for of the rickettsial gene gltA.

Tick species	frequency	Positive results (gltA)
*Rhipicephalus sanguineus* s.l	66,8 % (127/190)	3,1 % (4/127)
*Amblyomma ovale*	6,8 % (13/190)	7,7 % (1/13)
*Amblyomma aureolatum*	26,3 % (50/190)	8 % (4/50)

## 4 Discussion

To the author's knowledge, this is the first One Health approach of *Rickettsia* spp. exposure worldwide, with concomitant assessment in indigenous populations, their dogs and healthcare professionals. Although the human seropositivity found herein was lower than 5/34 (14.7 %) hunters of southern and central-western Brazil [4] and higher than 19/506 (3.8 %) patients during a Dengue outbreak in central-western Brazil [26], results of indigenous individuals (66/771; 8.6 %) and healthcare professionals (9/99; 9.1 %) were similar (*p* = 0.859) and may indicate similar exposure to ticks, regardless of living in indigenous communities.

No statistical differences were found herein between seropositivity and associated risk factors for human (living area, age, gender, hunting, tick bite history and dog owner) and dog exposure (age, sex, hunting under supervision, and living area). As previously shown for wild boar hunters in Brazil [27], the absence of statistical differences in the associated risk factors for indigenous may indicate a random exposure to *Rickettsia* spp. However, the tick bite history referred by healthcare professionals increased 9.29-fold the likelihood of *Rickettsia* spp. seropositivity, suggesting tick and *Rickettsia* spp. exposure during daily activities, associated to better capacity to recognize and report tick bites by healthcare professionals when compared to indigenous individuals. Tick bite history has also been reported in 55 %–60 % of Rocky Mountain Spotted Fever patients presenting illness onset, highlighting the importance of tick identification and prompt medical attendance [28]. Thus, health care education and awareness should be provided for indigenous individuals for prevention and early recognition of tick bites and tick-borne diseases in such vulnerable population. In the present study, correlation between seropositive dogs and positive ticks was not assessed due to low infection rate of *Rickettsia* spp. in ticks. The low infection rate has been previously reported under natural conditions and may be explained by lower survival and reproduction of infected tick populations [29,30]. In addition, further statistical analysis of owner-dog risk for *Rickettsia* spp. exposure was not possible because dogs of indigenous communities herein had outdoor access and shared multiple owners.

Despite dog exposure to rickettsial agents have been widely reported worldwide, few studies have focused on dogs living in indigenous communities. While only 3/130 (2.3 %) dogs living in a remote indigenous community of Australia were positive to *Rickettsia* spp. of SFG [31], 90/103 (87.4 %) dogs living in an indigenous community in Brazil were seropositive to *Rickettsia* spp., with 39/327 (11.9 %) infested by ticks and the highest reactivity to *R. amblyommatis* [32]. In this survey, *Rickettsia* spp. seropositivity was associated with age (adults), hunting, ethnicity and infestation of *Amblyomma* ticks [32]. In Brazil, dog seroprevalence herein (116/386; 30.1 %) was higher than 24/170 (14.1 %) hunting dogs from southern and central-western regions [4] and 62/ 282 (22.0 %) dog samples from traditional fishermen living in islands and seashore mainland of southern region [33], similar to 41/129 (31.7 %) dogs living in rural and urban areas of eastern Amazon [34], and lower than 16/25 (64.0 %) dogs from an endemic area of southern Brazil [35].

In the study herein, the bacteria *R. bellii*, not belonging to the SFG group, was considered the possible antigen involved in a homologous reaction in 71.5 % seropositive dog and 4.5 % human samples. Despite no statistical difference between homologous reaction was reported in humans, homologous antigenic reactions in dogs were significantly more frequent for *R. bellii* when compared to *R. parkeri* and *R. amblyommatis.* These results for dogs were very similar to 73.1 % of homologous reaction for *R. bellii* detected in a dog population from midwestern Brazil [36]. The high seroprevalence for *R. bellii* herein may be associated with the high diversity of hard and soft tick infection, with *R. bellii* reportedly able to infect and inhibit the maintenance of other pathogenic rickettsia in the Americas [37]. *Rickettsia bellii* infection has already been reported in *Amblyomma ovale* and *A. aureolatum* ticks, as reported herein of *Rickettsia* spp. infection not belonging to the SFG group [[38], [39], [40]]. Although reported in at least 25 tick species and may hinder vertical transmission of *Rickettsia* spp. belonging to the SFG, *R. bellii* has been generally considered as nonpathogenic for animals and humans [37].

Along with *R. rickettsii*, *R. parkeri* has been considered as an important pathogenic agent causing a milder spotted fever in Brazil, mainly transmitted by *Amblyomma ovale* in areas of the Atlantic rainforest biome [41]. Location of seropositive serum samples to *R. parkeri*, occurrence and positivity of *A. ovale* herein has corroborated with previous surveys, that have shown *R. parkeri* circulation in seashore areas of Paraná State in southern Brazil [33], Atlantic rainforest areas of São Paulo and Rio Grande do Sul States (37), and in southeastern and southern Brazil ([42,43].

Additionally, four (5.3 %) humans were seropositive or closely related genotype to *R. amblyommatis*, which remains of unclear human pathogenicity [44]. In the USA, seroconversion of *R. amblyommatis* and history of local rash following tick bite have been reported, along with febrile symptoms and erythema migrans after working in *Amblyomma americanum*-infested areas [44]. In addition, *R. amblyommatis* was identified in one *Rh. sanguineus* s.l. pool of Costa Rica and one *A. ovale* pool from Nicaragua, tick species found in the study areas [45].

## 5 Conclusions

To the author's knowledge, this is the first One Health approach worldwide to *Rickettsia* spp. exposure, with concomitant survey of indigenous populations, their dogs and healthcare professionals. The present study has suggested a high diversity of ticks and the circulation of different *Rickettsia* spp. in indigenous communities of southern and southeastern Brazil. The infection rates herein were lower than in other studied Brazilian areas and the most frequent *Rickettsia* species found in the present study have been considered non-pathogenic, with the lowest infection risk for populations. Nevertheless, these findings have highlighted the importance of health care education and awareness to indigenous individuals for prevention and early recognition of tick bites and tick-borne diseases in such communities in Brazil and worldwide.

## CRediT authorship contribution statement

**Louise Bach Kmetiuk:** Writing – review & editing, Writing – original draft, Methodology, Investigation, Data curation, Conceptualization. **Vamilton Alvares Santarém:** Writing – review & editing, Writing – original draft, Methodology, Investigation, Formal analysis, Conceptualization. **Daniele Rodrigues:** Investigation, Methodology, Writing – review & editing. **Suelen Teixeira de Faria Resende:** Investigation, Methodology, Writing – review & editing. **Isabella Braghin Ferreira:** Writing – review & editing, Methodology, Data curation. **Rogério Giuffrida:** Writing – review & editing, Writing – original draft, Methodology, Formal analysis, Data curation. **Bianca Bárbara Fonseca da Silva:** Writing – review & editing, Data curation. **Lucianne Cardoso Neves:** Writing – review & editing, Formal analysis. **Raphaela Bueno Mendes Bittencourt:** Writing – review & editing, Formal analysis. **Leandro Meneguelli Biondo:** Writing – review & editing, Methodology, Data curation. **Fabiano Borges Figueiredo:** Writing – review & editing, Investigation. **Felipe da Silva Krawczak:** Writing – review & editing, Writing – original draft, Methodology, Formal analysis, Data curation. **Alexander Welker Biondo:** Writing – review & editing, Writing – original draft, Visualization, Project administration, Methodology, Investigation, Funding acquisition, Conceptualization.

## Funding

The present research was funded through the Brazilian National Council for Scientific and Technological Development-CNPq (404687/2021-0).

## Declaration of competing interest

The authors declare that they have no known competing financial interests or personal relationships that could have appeared to influence the work reported in this paper.

## Data Availability

Data will be made available on request.
